# Neonatal Sacrococcygeal Fetiform Teratoma Containing Bowel: A Case Report

**DOI:** 10.1055/a-2206-4825

**Published:** 2024-01-22

**Authors:** Ashley Toms, Tarlia Rasa Govender, Giulia Brisighelli, Derek Harrison

**Affiliations:** 1 Department of Paediatric surgery, Chris Hani Baragwanath Academic Hospital, Faculty of Health Sciences, University of Witwatersrand, Johannesburg, South Africa

**Keywords:** sacrococcygeal teratoma, fetiform teratoma, fetus-in-fetu, germ cell tumor

## Abstract

A fetiform sacrococcygeal teratoma (homunculus) is a highly differentiated subgroup of mature cystic teratoma that resembles a malformed fetus. These tumors originate at the base of the coccyx and may vary in their intrapelvic and extrapelvic extent and location. It is important to differentiate this anomaly from fetus-in-fetu which has a higher degree of structural organization. A 5-day-old neonate presented with a type II sacrococcygeal fetiform teratoma. The mass contained both cystic and solid components. Upon surgical excision and coccygectomy, fully formed bowel was found inside the mass, as well as bones and other well-defined structures. The tumor was confirmed to be fully excised and no malignant or immature features were found on histopathological examination. The patient was last seen growing well with an alpha-fetoprotein of 3.5 μg/L, 14 months after resection.

## Introduction

Sacrococcygeal teratoma (SCT) is a germline tumor arising at the base of the coccyx. 1 It usually contains tissue from all three primordial germ layers; ectoderm, mesoderm, and endoderm. 1 2 It is the most common type of congenital germline tumor with a male-to-female ratio of 1:4. 1 2 The most common sites for childhood extragonadal teratomas are sacrococcygeal, mediastinal, retroperitoneal, and the central nervous system. 1 2 A fetiform teratoma (homunculus) is a rare, highly differentiated subgroup of a mature cystic teratoma that resembles a malformed fetus. Fetiform teratomas have potential for malignant transformation and an independent ability for growth. 3 4 It is important to distinguish this anomaly from fetus-in-fetu (FIF), a monozygotic twin with a higher degree of structural organization commonly found in the retroperitoneum in neonates. 5 In resourced settings, the diagnosis is usually made antenatally with routine obstetric ultrasonography. It is also used to determine risk to fetal survival, the need for fetal therapy, and mode and timing of delivery. 6 Early surgical resection and complete coccygectomy after the first few days of life is the cornerstone of management for fetiform teratomas. 7 This case report can be distinguished from other fetiform teratomas found in literature due to the age of presentation and the very high degree of organ differentiation. It also provides an opportunity to clarify the differences between fetiform teratomas and FIF and to assist in reaching the correct diagnosis.

## Case Report

**Fig. 1:**
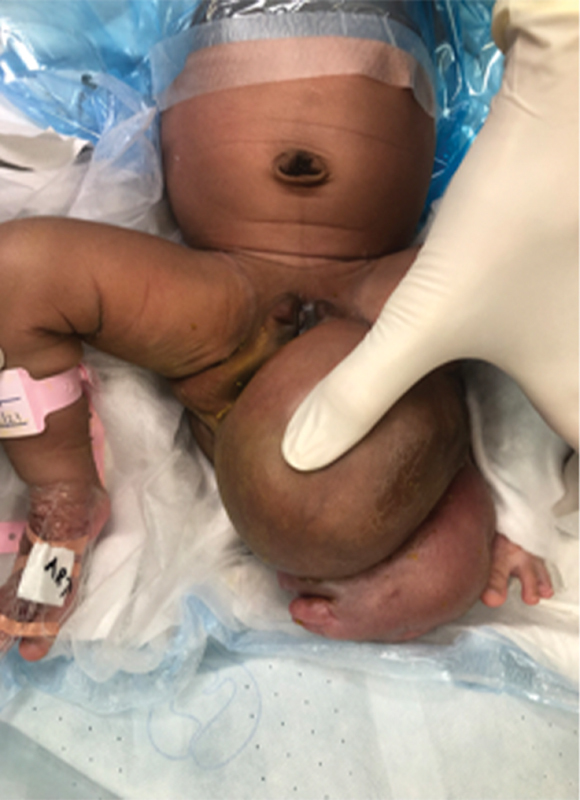
Preoperative photo of the patient showing the anterior view of the multilobulated mass.

**Fig. 2:**
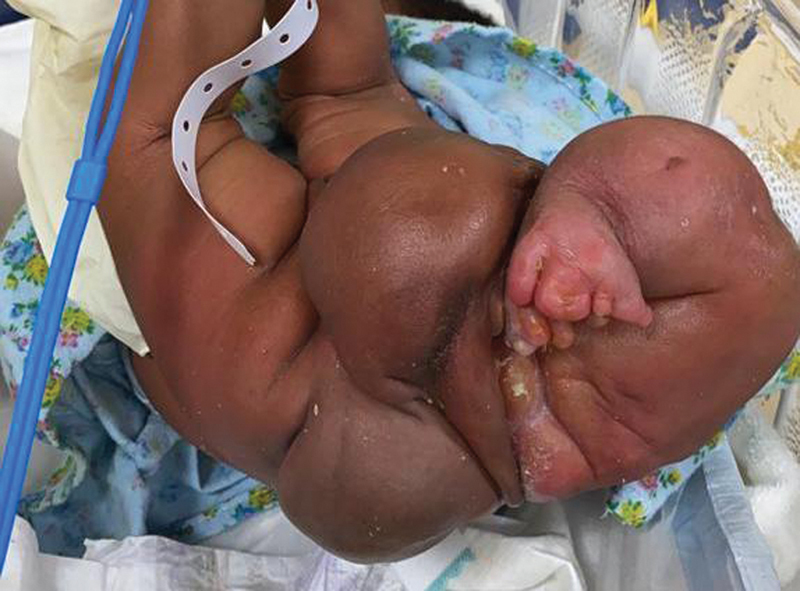
Posterior view of the mass with homunculus features emanating from the right buttock.

A 3,940-g baby girl was delivered via cesarean section at 39 weeks' gestation with good Apgar scores at a periphery hospital. Her mother had an uneventful pregnancy with no antenatal imaging performed. At birth, a large sacral mass was observed and she was transferred to our institution on day 5 of life for pediatric surgical care. On arrival the patient was noted to have normal hemodynamics, had been breastfeeding, and passing stool and urine without difficulty. She had no facial dysmorphic features. On examination of her back and perineum, she was noted to have a prominent multilobulated mass extending from her midline and right buttock resembling malformed lower limbs and feet ( Figs. 1 and 2 ). Anterior to the mass, normal external genitalia and anus was observed.

**Fig. 3:**
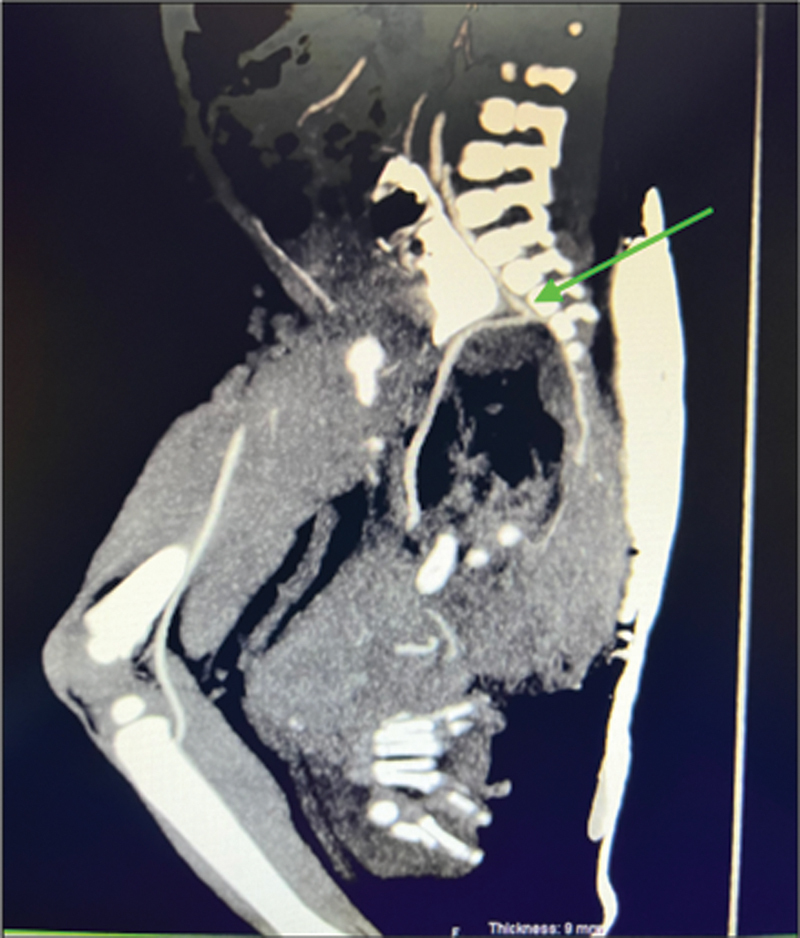
Sagittal view showing a prominent middle sacral artery supplying the mass.

The hematological and serological investigations showed a normal full blood count, an alpha-fetoprotein (AFP) of 24,504 μg/L (within normal range), and a beta-human choriogonadotropin of less than 1. The computed tomography (CT) scan of the abdomen and pelvis revealed a sacrococcygeal mass between the coccyx and the lower sacral vertebrae with both intra- and extrapelvic extension. The mass was both cystic and solid in nature and measured 7.0 × 11.8 × 11.4 cm. A large middle sacral artery was demonstrated by trifurcation of the aorta ( Fig. 3 ). The CT also showed an accessory femur, multiple metatarsal bones, and phalanges. There was no bladder, bowel, or intra-abdominal abnormalities noted. The teratoma was classified as Altman type II by the American Academy of Pediatric Surgical Section classification.

## Surgical Management

**Fig. 4:**
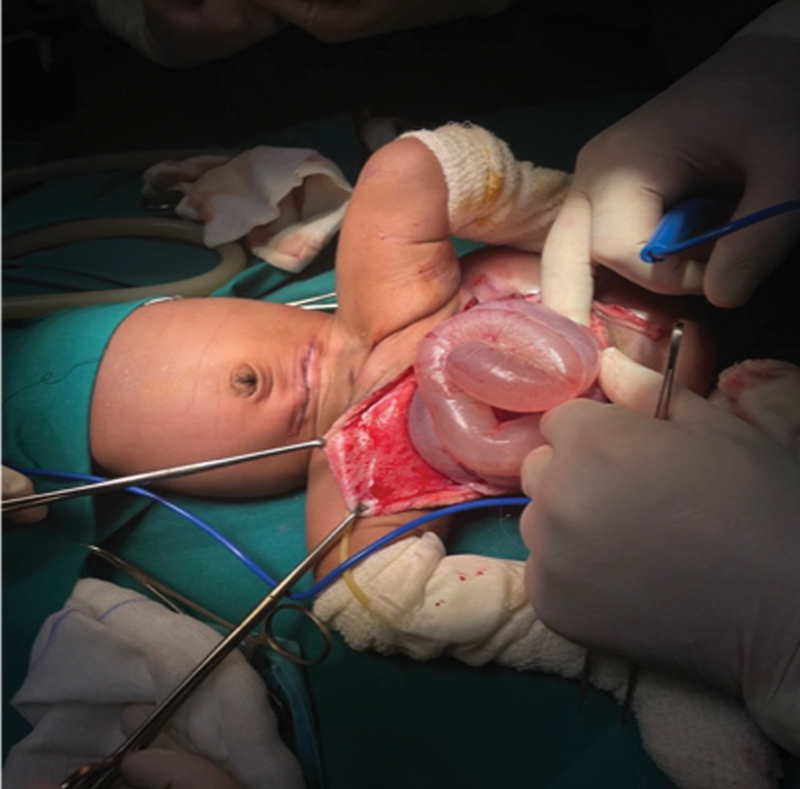
Intraoperative cystic rupture revealed bowel content.

**Fig. 5:**
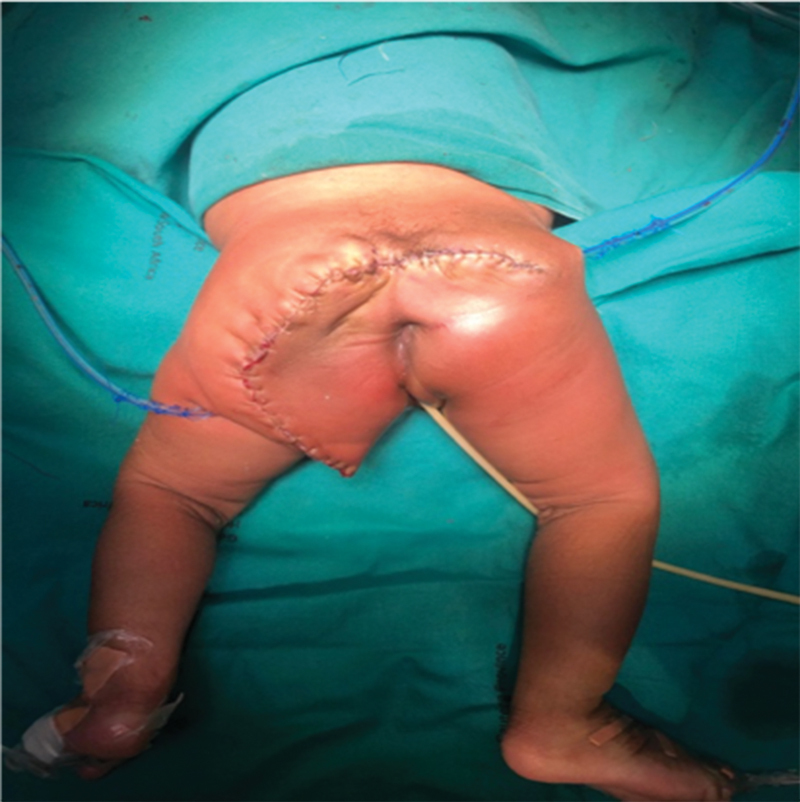
Post-mass resection and reconstruction.

Surgical resection was undertaken on day 12 of life under general anesthesia. The patient was placed in a supine position and a Pfannenstiel incision was made. The supplying artery originating from the abdominal aorta was identified, ligated, transected, and the abdomen closed. Exposure was initially gained on the anterior aspect of the teratoma ( Fig. 4 ). Dissection followed to separate the mass from the skin and the gluteal muscles. The neonate was then placed in a prone position for further dissection and the mass was freed from the rectum. Every effort was made to prevent intraoperative cystic rupture, but rupture was unavoidable. However, despite rupture, there was no spillage into any body cavity as the tumor ruptured on the outside. The tumor and coccyx were then completely excised en bloc. The skin was closed over two negative pressure drains. An adequate cosmetic result was achieved ( Fig. 5 ). Postoperatively, the child was transferred to the neonatal intensive care unit in a stable condition. Histological examination of the mass showed epidermis, dermis, and subcutaneous tissue and proliferation of fibro-adipose tissue, intestinal mucosa, and pancreatic parenchyma. The attached bowel showed mucosal erosion, congestion, and submucosal edema. Hyaline cartilage was present as nodules. The cartilage showed endochondral ossification in keeping with the coccyx. The immature digits inside of the mass comprised of central cartilage and bone. Additionally, there were numerous nerve fibers containing ganglionated cells with no immature components or malignant transformations. The cystic component of the mass contained bowel with a good vascular supply. These findings were compatible with the features of a benign fetiform teratoma. Follow-up AFP at 14 months postresection was 3.5 μg/L, indicating that there is no recurrence and a low potential for malignancy.

## Discussion

There are various theories describing the origin of teratomas. These theories include parthenogenetic development of germ cells within the gonads or in extragonadal sites; nonparthenogenetic development from “wandering germ cells” that were left behind during the migration of embryonic germ cells from the yolk sac to the gonad; and development from other totipotent embryonic cells. 8 The most popular theory for SCTs suggests that teratomas originate from Henson's node, a cluster of pluripotent stem cells, located near the coccyx. 2 Germ cell tumors are classified histologically as teratomas, which can either be mature, immature, or malignant. 2 9 Immature teratomas consist of incomplete differentiated tissue structures of which primitive neural tubes and rosettes are commonly found. 9 The grade of immaturity is best assessed according to the Gonzales–Crussi classification. This method is used to assess the amount of immature neural structures found in teratomas. 10

A mature teratoma is more commonly found and consists of fully differentiated tissue originating from a variety of somatic sites. A mature teratoma can contain fully developed bone structures, glandular structures, or even a fully developed intestinal segment. 9 Rarely, a mature teratoma develops a high degree of differentiation and complex tissue organization, taking the shape of a malformed fetus. 5 This entity is known as a fetiform teratoma or “homunculus”—Latin for little man. A bony skeleton is often present with varying degrees of limb formation; however, visceral organ tissue and skeletal muscle are generally undistinguished or absent. 5 It is associated with better caudal differentiation than cephalic. 11

The main differential for a fetiform teratoma is FIF. 3 5 11 This is a rare anomaly in a diamniotic monochorionic twin pregnancy where the parasitic twin is commonly found retroperitoneally in the body of the other twin. 3 5 It is postulated that this is due to unequal division of totipotent inner cells of the blastocyst, with inclusion of these cells into the more mature embryo. 5 Another theory suggests a persistent anastomosis of the vitelline circulation, causing one fetus to merge into the other during the second and third week of gestational development. 3 5 This anomaly is found more commonly in males with an incidence of 1:500,000 births. 3

FIF has characteristically been distinguished from fetiform teratoma by the presence of a segmented axial skeleton. 3 5 This shows that the fetus has went through a primary stage of gastrulation, involving formation of the neural tube. 4 Another differentiating feature of FIF is the presence of highly developed organ systems, while fetiform teratoma usually does not consist of complex visceral differentiation. FIF is also invariably associated with anencephaly and acardia. 5 11 However, organ differentiation in fetiform teratoma has been reported in the literature. Most notably, Kuno et al, who reported a highly developed axial skeleton and organ development in an ovarian fetiform teratoma. 12 In our case, a mature developed segment of bowel was found within the teratoma. A fetiform teratoma presents most commonly in the ovaries of women at reproductive age in the 3rd to 4th decade of life. 5 FIF usually presents retroperitoneally in infancy suspended by a pedicle encapsulated in a well-defined sac-like remnant of the amniotic sac. 3 4 FIF and fetiform teratoma can also be diagnostically differentiated based on zygosity. 5 A teratoma is generally homozygous at the loci while the host tissue is heterozygous. On the other hand, FIF is genetically identical to the host and may be viewed by some as a variation of conjoined twinning. 5 Cytogenetic examination was not performed in our case.

Differentiating the two entities is important as FIF is more commonly benign, while a fetiform teratoma has a 10% chance for malignant transformation ( Table 1 ). 4 However, it has been suggested in the literature that FIF and fetiform teratoma rather exist on a spectrum of the same pathology both at different stages of maturation. 3 4 In this case, the mass extended from the coccyx and was not found to be retroperitoneal as commonly seen in FIF. Due to the absence of an axial skeleton and the presence of a bony skeleton with varying degrees of limb formation, the mass has been concluded to be a fetiform teratoma.

**Table 1:** Comparison between Fetiform Teratoma and Fetus-in-fetu

	Fetiform teratoma	Fetus-in-fetu
Definition	• Also known as homunculus, Latin for ‘‘little man’' 3 4 • Rare form of teratoma that resembles a malformed fetus 3 4 • Well organized and highly differentiated mature cystic teratoma with organoid differentiations 3 4	• Rare anomaly in a diamniotic monochorionic twin pregnancy where the parasitic twin is commonly found retroperitoneally in the body of the other twin 3 5
Location	• Most commonly discovered as ovarian masses 5	• The most common location is retroperitoneal 3 4
Characteristics	• Bony skeleton is typically present • Varying degrees of limb formation, but visceral organ tissue and skeletal muscle are generally inconspicuous or absent 5	• Highly developed and segmented axial skeleton 3 5 • Almost always presents with acardia and anencephaly 5 11
Pathogenesis	• Thought to arise from parthenogenetic development of a primordial germ cell 5 • Mature cystic teratomas arise from a single germ cell that has completed the first meiotic division 5	• Thought to be due to unequal division of totipotent inner cells of the blastocyst, with inclusion of these cells into the more mature embryo 5 • Thought to be associated with persistent anastomosis of the vitelline circulation, causing one fetus to merge into the other during the 2nd and 3rd week of gestational development 3 5
Age distribution	• Most commonly found in women of reproductive age 5	• Most reported cases have been discovered in infancy as an abdominal mass 3 4
Zygosity	• Commonly homozygous at loci, but the host normal tissue indicates heterozygosity 5	• FIF is genetically identical to its host 5
Malignant potential	• 10% chance for malignant transformation 4	• More commonly benign 4
Management	• Measuring biochemical tumor markers to detect potential malignancy 1 • Surgical excision of the mass 2 7 8 • Follow-up regularly as recurrences are likely to appear in the first 3 years of life 1 6	• Tumor marker surveillance 4 • Surgical excision of mass has reported good outcomes 4 • Minimum of 2-year follow-up to prevent a missed malignancy 4

Abbreviation: FIF, fetus-in-fetu.

Despite successful surgical excision of the mass, the development of urinary incontinence (24–30%), soiling (13–29%), constipation (17–47%), involuntary bowel movements (9–12%), and total fecal incontinence (7–12%) are the most commonly observed complications of surgery. 7 13 Thus, appropriate bowel and bladder functional assessments should be done at subsequent follow-ups to detect any signs of these complications. Incomplete resections and tumor rupture are commonly linked with recurrence. Ten to 20% of recurrences will appear during the first 3 years of life, thus it is important to follow-up regularly by checking AFP levels and doing rectal examinations every 3 months for the first 2 years and then every 6 months until 5 years of age. 1 6

## Conclusion

A fetiform SCT is a rare variation of SCTs. This phenotypically bizarre condition must be differentiated from FIF. As with all SCTs, tumor markers, correct imaging, and planning allow for appropriate surgical excision to be performed soon after birth. Continued surveillance for malignancy is essential postoperatively. Finally, long-term, cosmetic results and appropriate bowel and bladder functional assessments are important when age appropriate.
